# Pilot Evaluations of Two Bluetooth Contact Tracing Approaches on a University Campus: Mixed Methods Study

**DOI:** 10.2196/31086

**Published:** 2021-10-28

**Authors:** Tyler Shelby, Tyler Caruthers, Oren Y Kanner, Rebecca Schneider, Dana Lipnickas, Lauretta E Grau, Rajit Manohar, Linda Niccolai

**Affiliations:** 1 Epidemiology of Microbial Diseases Department Yale School of Public Health Yale University New Haven, CT United States; 2 Yale School of Medicine Yale University New Haven, CT United States; 3 Information and Technology Services Yale University New Haven, CT United States; 4 Yale School of Engineering and Applied Science Yale University New Haven, CT United States

**Keywords:** mHealth, digital contact tracing, Bluetooth, COVID-19, mixed methods

## Abstract

**Background:**

Many have proposed the use of Bluetooth technology to help scale up contact tracing for COVID-19. However, much remains unknown about the accuracy of this technology in real-world settings, the attitudes of potential users, and the differences between delivery formats (mobile app vs carriable or wearable devices).

**Objective:**

We pilot tested 2 separate Bluetooth contact tracing technologies on a university campus to evaluate their sensitivity and specificity, and to learn from the experiences of the participants.

**Methods:**

We used a convergent mixed methods study design, and participants included graduate students and researchers working on a university campus during June and July 2020. We conducted separate 2-week pilot studies for each Bluetooth technology. The first was for a mobile phone app (“app pilot”), and the second was for a small electronic “tag” (“tag pilot”). Participants validated a list of Bluetooth-identified contacts daily and reported additional close contacts not identified by Bluetooth. We used these data to estimate sensitivity and specificity. Participants completed a postparticipation survey regarding appropriateness, usability, acceptability, and adherence, and provided additional feedback via free text. We used tests of proportions to evaluate differences in survey responses between participants from each pilot, paired *t* tests to measure differences between compatible survey questions, and qualitative analysis to evaluate the survey’s free-text responses.

**Results:**

Among 25 participants in the app pilot, 53 contact interactions were identified by Bluetooth and an additional 61 by self-report. Among 17 participants in the tag pilot, 171 contact interactions were identified by Bluetooth and an additional 4 by self-report. The tag had significantly higher sensitivity compared with the app (46/49, 94% vs 35/61, 57%; *P*<.001), as well as higher specificity (120/126, 95% vs 123/141, 87%; *P*=.02). Most participants felt that Bluetooth contact tracing was appropriate on campus (26/32, 81%), while significantly fewer participants felt that using other technologies, such as GPS or Wi-Fi, was appropriate (17/31, 55%; *P*=.02). Most participants preferred technology developed and managed by the university rather than a third party (27/32, 84%) and preferred not to have tracing apps on their personal phones (21/32, 66%), due to “concerns with privacy.” There were no significant differences in self-reported adherence rates across pilots.

**Conclusions:**

Convenient and carriable Bluetooth technology may improve tracing efficiency while alleviating privacy concerns by shifting data collection away from personal devices. With accuracy comparable to, and in this case, superior to, mobile phone apps, such approaches may be suitable for workplace or school settings with the ability to purchase and maintain physical devices.

## Introduction

### Background

Following its identification in Wuhan, China in December 2019, SARS-CoV-2 rapidly spread across the globe, resulting in millions of infections and deaths due to COVID-19 [1]. As health organizations throughout the world worked to develop adequate pharmaceutical therapies and vaccines, many public health agencies relied on nonpharmaceutical interventions to reduce community transmission of SARS-CoV-2. In particular, the world relied on mass screening [2], lockdowns [2], physical distancing [3], mask wearing [4], and contact tracing [5]. While large-scale lockdowns and comprehensive masking interventions are less commonly seen in public health interventions, contact tracing is a traditional intervention that has proven effective in many other contexts [6-8]. However, the implementation of contact tracing for SARS-CoV-2 has faced many challenges due to high incidence rates, even among asymptomatic individuals [9], presymptomatic transmission [10], and, in many places, a lack of staffing and infrastructure [11]. These challenges made it difficult in many settings to achieve the yield (proportion of cases and contacts interviewed, isolated, and/or quarantined) and timeliness (time from symptom onset or testing to isolation for cases, and time from exposure to quarantine for contacts) thought to be required for effectiveness [12,13].

These challenges shifted the focus of many health agencies to mitigation (rather than containment) and led many to propose contact tracing innovations designed to make tracing more feasible [14]. While traditional contact tracing relies on interviewing cases and contacts in-person or by telephone, several countries augmented data collection using individual-level GPS data [15], Bluetooth technology [16], and other personalized data sources [17]. One technology in particular, Bluetooth, gained widespread attention in both the press [18] as well as scientific literature [19]. Despite the theoretical benefits of Bluetooth-assisted contact tracing and its implementation in various countries [16], the public health and lay communities are far from reaching consensus regarding the appropriateness [20] and effectiveness [21,22] of this innovation, largely due to 2 reasons.

First, many have raised concerns about the loss of individual privacy associated with automated data collection methods such as Bluetooth-assisted tracing [23,24]. In many countries, mandating participation in Bluetooth-assisted contact tracing is not feasible, and the effectiveness of this approach relies on a high user uptake among the population [22]. Implementation of Bluetooth-assisted tracing apps in nonmandated settings has so far been met with low uptake [25,26], and therefore, a better understanding of potential users’ perceptions and privacy concerns is needed. Second, while research in other contexts has found various technologies, including radio frequency detectors, Wi-Fi, and Bluetooth, to be helpful in the detection of contact interactions [27-29], there are few studies evaluating the overall impact and effectiveness of Bluetooth-assisted tracing in the context of COVID-19 [30,31]. Although it seems intuitive that Bluetooth-assisted data collection may lead to an increase in the total number of identified COVID-19 “close contacts” (defined by the Centers for Disease Control and Prevention [CDC] as in-person interactions within 6 feet for at least 15 minutes) and more rapid identification of these individuals, there is little real-world data to directly verify this or to evaluate the accuracy of Bluetooth data [21,30].

### Goal of This Study

Together, doubts about the appropriateness and acceptability of Bluetooth-assisted contact tracing and the accuracy and reliability of the data pose challenges to implementation and adoption. Due to low vaccine uptake [32,33] and breakthrough transmission by variant strains [34], overcoming these challenges is critical as contact tracing will remain a core part of the public health response to COVID-19, even in the postvaccine phase of the pandemic. To address these knowledge gaps, we pilot tested 2 different Bluetooth-assisted tracing technologies on a university campus, one which collected Bluetooth data using a mobile phone app and another that used a separate carriable device (“tag”) with Bluetooth functionality. Using a convergent mixed methods design, we measured the sensitivity and specificity of each Bluetooth technology and assessed participant perceptions regarding appropriateness, usability, acceptability, and adherence, using a quantitative survey and qualitative free-text analysis.

## Methods

### Study Setting and Population

We conducted 2 separate pilot studies in June to July 2020 at a medium-sized private university in the US Northeast. During this time, only essential personnel and select individuals were allowed on campus with prior approval. Campus-wide precautions included mask wearing, physical distancing, daily symptom assessments, and testing. Study participants included graduate students and researchers working during this period; graduate students or researchers working from home were ineligible for participation. We recruited participants by emailing faculty members and lab supervisors who subsequently forwarded our recruitment emails to their students and research staff. We then selected labs with the highest acceptance rates. We also prioritized enrollment from labs that shared workspaces with other recruited labs. Due to the focused nature of the pilots, we did not collect demographic data from participants. Each of the sequential pilots lasted 2 weeks (14 days) starting on a Monday, and different labs participated in the separate pilots. Sample size was determined by the availability of required study devices. The collected data were stored on secure university servers throughout the study and analysis period.

### Pilot 1: Mobile Phone–Based Bluetooth

#### Technology

In the first pilot (hereafter referred to as the “app pilot”), we evaluated a mobile phone app developed by the university’s information technology services staff (Multimedia Appendix 1). It functioned by detecting Bluetooth signals emitted by other phones that had the same app downloaded and activated. The app estimated the distance between mobile phones based on signal strength while recording the duration of the interaction. The app also had functionality for users to enter a date of symptom onset or positive test; however, this function was not used during the pilot. Data were automatically sent to a centralized server. The university provided Android phones to participants for the duration of the study, so that they did not have to download the app on their personal devices.

#### Setting and Data Collection

All app pilot participants were provided with written instructions describing how to install and use the mobile app and how to validate and report new contact interactions, as well as contact information for technical support if needed. Participants were asked to carry the study phone while on campus. At the end of each day, participants reviewed an online spreadsheet of their Bluetooth-identified close contacts and confirmed or denied each interaction. We also asked participants to identify additional contacts that were not detected by Bluetooth, and we subsequently removed any self-reported contacts who were not study participants. Participants were asked to use their best judgment when estimating the length of each interaction.

### Pilot 2: Tag-Based Bluetooth

#### Technology

In the second pilot (hereafter referred to as the “tag pilot”), we evaluated a carriable device (“tag”) equipped with Bluetooth functionality, designed by the author RM (Multimedia Appendix 2 and Multimedia Appendix 3). The tags recorded Bluetooth signals emitted from other tags, using signal strength to determine distance while recording the duration of interactions. Data were stored locally on the tags and routinely synced to a central server by study participants using a mobile app that paired with the participant’s tag. The app only used Bluetooth to communicate with the tag while syncing and otherwise did not collect any additional data or use Bluetooth to communicate with any nonpaired tags or other devices. The tag software additionally allows for contact interactions to be encrypted when recorded and stored in the central server, thereby anonymizing the data. When this feature is active, decrypting the data requires the user to provide permission by submitting a decryption token through the app. However, this feature was not enabled during the study, so that we could determine all contact records for the purpose of evaluating the system’s efficacy. Additional details regarding the tag’s development can be found elsewhere [35]. The university provided participants with Android phones for the duration of the pilot to facilitate syncing of tag data. Participants were asked to use their best judgment when estimating the length of each interaction.

#### Setting and Data Collection

All tag pilot participants were provided with written instructions describing how to install and use the mobile syncing app, how to pair it with their Bluetooth tag, and how to validate and report new contact interactions, as well as contact information for technical support if needed. Participants were asked to carry the tag while on campus and to sync their Bluetooth data after each shift. At the end of each day, participants reviewed a list of their Bluetooth-identified close contacts and confirmed or denied each interaction using an online web interface. We also presented participants with the estimated duration of each recorded interaction and asked participants to report if the duration was underestimated or overestimated. Similar to the app pilot, we asked participants to identify additional contacts not detected by Bluetooth and subsequently removed those who were not study participants.

### Postparticipation Survey

Following each pilot, we sent a survey to participants focusing on their experiences using the pilot technology, as well as their perceptions regarding the appropriateness of technology-assisted tracing on campus (see Table 1 for survey domains). We adapted this survey from a previously validated mHealth usability questionnaire [36]. Most questions used a 7-point Likert scale ranging from *strong agreement* to *strong disagreement*, including a *neutral* response option. The survey also contained a free-text question asking participants to provide any additional comments about their experience or suggestions about the technology. We used Cronbach alpha to measure the reliability of our adapted scale after aligning the directionality of question responses. We excluded the free-text response and 2 other scale items from the reliability measurement that asked participants to select various ways in which they carried the devices or reasons why they were not carried.

**Table 1:** Postparticipation survey overview.

Domain and subdomain		Goals within the domain/subdomain
Appropriateness		To measure participant perceptions about the appropriateness of Bluetooth contact tracing and the use of certain types of data (Bluetooth, GPS, Wi-Fi, etc)
**Usability**		
	Ease of use	To measure the ease with which participants install, learn to use, and use the apps
	Interface and satisfaction	To measure participant experiences and satisfaction with the design and interface of the app
**Acceptability**		
	Usefulness	To evaluate participant beliefs surrounding the usefulness of the tracing technology
	Coherence	To evaluate participants’ understanding of how data are collected and protected by the technology
	Social influence	To measure the presence of social influence from peers or supervisors regarding uptake of technology-assisted tracing
	Setting	To measure perceptions about available assistance for the use of the apps and/or devices and individual agency in uptake
Adherence		To measure adherence and participant preferences with regard to carrying the study devices

### Analysis Plan

#### Quantitative Study Outcomes and Measurements

We used participants’ daily contact validation responses to estimate the sensitivity and specificity of the 2 technologies (see Table 2 for outcome and measure definitions) and used 2-tailed tests of proportions to compare these values between pilots. We also described the postparticipation survey by presenting proportions of participants agreeing with each Likert question or selecting responses from other categorical questions, as well as means for responses to continuous questions. We measured differences in survey responses between participants from different pilot groups using 2-tailed tests of proportions for Likert agreement and categorical questions, and unpaired 2-tailed *t* tests for continuous questions. Additionally, we used paired tests of proportions to measure differences between agreement with several comparable survey questions, including (1) appropriateness of Bluetooth vs location data (GPS and/or Wi-Fi) for contact tracing, (2) peer vs supervisor vocal support of study technology, and (3) peer vs supervisor vocal concern about the study technology.

**Table 2:** Definitions of Bluetooth measures and outcomes.

Measures/outcomes			Definition
**Measures^a^**			
	True positive	Bluetooth-identified contact that is confirmed by the participant	
	True negative	No contacts detected, confirmed by the participant	
	False positive	Bluetooth-identified contact denied by the participant	
	False negative	Participant-recalled contact that was not detected by Bluetooth	
**Outcomes**			
	Sensitivity	True positive/(true positive + false negative)	
	Specificity	True negative/(true negative + false positive)	

^a^15 minutes of interaction within 6 feet required to meet the definition of “close contact.” In addition to confirming/denying each close contact interaction, participants from the tag pilot were asked to comment on the underestimation or overestimation of the recorded contact duration. We allowed a 5-minute window of error, within which a contact’s measurement type could be altered. For example, a contact detected for 15-19 minutes would be designated as a false positive if the study participant noted that the interaction length was overestimated, while a contact detected for 10-14 minutes would be designated as a false negative if the study participant noted that the interaction length was underestimated.

#### Qualitative Analysis of Free-Text Responses

The coding team (TS and LG) used a codebook that was deductively based upon the survey topics. TS coded the free-text responses, and the coding team met regularly to review the coded text and reach agreement on all coding decisions. The coding team also refined code definitions and generated new codes when applicable throughout the coding process. “RADaR,” a rapid qualitative analysis approach [37], was used, in which the coding and analysis were done in Microsoft Excel (Microsoft Corp) rather than in a traditional qualitative analysis software. We synthesized the qualitative and quantitative aspects as part of the mixed methods analysis [38,39] by identifying quotes that provided greater context or deeper understanding for the findings from the quantitative survey analyses. Selected quotes are presented alongside the quantitative findings within the relevant survey domains.

### Study Approval

This study was approved by the Yale Human Subjects Committee, and written consent was obtained from participants prior to enrollment. We did not offer incentives for participation.

## Results

### Study Participants, Number of Shifts, and Frequencies of Contact Interactions

We invited 33 participants from 7 labs for the app pilot, of which 30 agreed to participate, and 25 completed the 2-week period of follow-up. Overall, 53 contact interactions were identified via Bluetooth, and an additional 61 were reported by participant recall. We invited 24 participants from 2 labs for the tag pilot, of which 17 agreed to participate, and all completed the 2-week period of follow-up. A defect was identified in the tag cases at the end of the first week of data collection that rendered the data unusable. The cases were then replaced, and only the data from the second study week were further analyzed. In the second week of data collection, 171 contact interactions were identified by Bluetooth and an additional 4 were reported by participant recall.

### Sensitivity and Specificity

We present estimates of sensitivity and specificity, and counts of true/false positives and negatives in Table 3, stratified by pilot. The tag pilot had significantly higher sensitivity compared to the app pilot (46/49, 94% vs 35/61, 57%; *P*<.001), as well as higher specificity (120/126, 95% vs 123/141, 87%; *P*=.02). Of note, 3 participants in the tag pilot reported leaving their tags on their desks during days on which they were not on campus, resulting in false recordings of contact interactions. When these interactions were removed from the data set, sensitivity and specificity became 93% (43/46) and 100% (111/111), respectively.

**Table 3:** Counts of true/false positives and negatives, and estimates of sensitivity and specificity.

Measures/outcomes			App pilot		Tag pilot
**Measures, n**					
	True positive	35		46	
	True negative	123		120	
	False positive	18		6	
	False negative	26		3	
**Outcomes, %**					
	Sensitivity	57%		94% (93%^a^)	
	Specificity	87%		95% (100%^a^)	

^a^Adjusted values after removing erroneous contact records from tags left on participants’ desks when they were not on campus.

### Postparticipation Survey

Twenty participants from the app pilot and 12 participants from the tag pilot completed the postparticipation survey (Cronbach α=.90). Below, we present the quantitative results from each section alongside qualitative findings when applicable.

#### Appropriateness

Overall, there were no differences in perceived appropriateness of technology-assisted tracing among participants between pilot groups (Table 4). Most participants felt that contact tracing via Bluetooth was appropriate but felt that the use of additional location data such as GPS or Wi-Fi was less appropriate (26/32, 81% approval for Bluetooth vs 17/31, 55% approval for GPS/Wi-Fi; *P*=.02). Most participants also preferred technology developed and managed by the university rather than a third party (27/32, 84%) and preferred to not download apps on their personal devices (21/32, 66%).

Regardless of the approach, most participants (24/32, 75%), though not all, reported concerns about how their privacy would be protected, and these concerns were expanded upon in the free-text data.

One [lab member] voiced concerns about how individual GPS contact data might be used against individuals (such as by police in the case of protests) - sadly, similar to what actually happened with a Mayor releasing names publicly recently....I think if the privacy aspect is addressed VERY clearly and intentionally it might increase the acceptance.

App pilot, Participant #3

I do have some concerns with privacy, but I am not sophisticated enough in this topic to articulate my concerns or to understand if I should be concerned or not. I think the data from a school-wide system does have the potential to be abused, but I think an effective contact tracing system should/could significantly increase the safety of students, faculty, and staff on campus.

Tag pilot, Participant #17

**Table 4:** Postparticipation survey: appropriateness domain.

Questions	Total percentage agreement^a^ (N=32)^b^, % (n/N)	App percentage agreement^a^ (N=20)^b^, % (n/N)	Tag percentage agreement^a^ (N=12)^b^, % (n/N)	*P* value^c^
It is appropriate for the university to use Bluetooth apps to monitor interactions on campus in order to more efficiently perform contact tracing.	81 (26/32)	80 (16/20)	83 (10/12)	.82
It is appropriate to use location information such as GPS and Wi-Fi connection data for contact tracing.	55 (17/31)	58 (11/19)	50 (6/12)	.67
I would prefer to use a contact tracing app on a university-owned device as opposed to downloading the app on my personal phone.	66 (21/32)	65 (13/20)	67 (8/12)	.92
I would prefer to use an app developed and owned by the university as opposed to an app developed and owned by an independent third party.	84 (27/32)	85 (17/20)	83 (10/12)	.90
I have concerns about how using this app, or an app like it, could affect my privacy.	75 (24/32)	70 (14/20)	83 (10/12)	.40

^a^Percentage agreement was calculated by dividing the number of Likert responses indicating agreement by the total number of Likert responses for each question.

^b^Some questions were not answered by all participants; exact counts of agreement and total responses are shown in parentheses for each question.

^c^*P* values obtained using tests of proportions to evaluate differences between pilots.

#### Usability

There were no observed differences between pilot groups regarding app usability (Table 5), and most participants from both pilots felt their respective apps were easy to install (25/31, 81%) and use (31/32, 97%). They also reported moderate levels of satisfaction with the app interfaces (21/32, 66%) and feedback from the apps (18/31, 58%). The amount of time required to use the apps was acceptable to most (29/32, 91%), and overall satisfaction was high (26/32, 81%). However, several participants from both pilots described difficulties downloading and installing the apps, syncing tags to mobile devices for uploading data, discerning how the app was responding to the user due to unclear feedback from the app, or experiencing other technological glitches.

[The app] would switch tracking off by itself.

App pilot, Participant #13

When I first obtained the phone, there was no contact tracing app on it, and I could not find a way to download it…When I tried syncing the tag to the phone, there was never a message telling me that the tag was synced, only “connecting” and “communicating.”

Tag pilot, Participant #19

**Table 5:** Postparticipation survey: usability domain.

Subdomains and questions			Total percentage agreement^a^ (N=32)^b^, % (n/N)		App percentage agreement^a^ (N=20)^b^, % (n/N)		Tag percentage agreement^a^ (N=12)^b^, % (n/N)		*P* value^c^
**Ease of use**									
	It was easy for me to install the app on the device.	81 (25/31)		84 (16/19)		75 (9/12)		.53	
	It was easy for me to learn to use the app.	97 (31/32)		95 (19/20)		100 (12/12)		.43	
	The app was easy to use.	97 (31/32)		95 (19/20)		100 (12/12)		.43	
**Interface and satisfaction**									
	I like the interface of the app.	66 (21/32)		65 (13/20)		66 (8/12)		.92	
	The information in the app was well organized, so I could easily find the information I needed.	71 (22/31)		63 (12/19)		83 (10/12)		.23	
	The app adequately acknowledged and provided information to let me know the progress of my action.	58 (18/31)		53 (10/19)		66 (8/12)		.44	
	The amount of time involved in using the app is acceptable.	91 (29/32)		85 (17/20)		100 (12/12)		.16	
	I would use this system again.	78 (25/32)		70 (14/20)		92 (11/12)		.15	
	Overall, I am satisfied with this system.	81 (26/32)		80 (16/20)		83 (10/12)		.82	

^a^Percentage agreement was calculated by dividing the number of Likert responses indicating agreement by the total number of Likert responses for each question.

^b^Some questions were not answered by all participants; exact counts of agreement and total responses are shown in parentheses for each question.

^c^*P* values obtained using tests of proportions to evaluate differences between pilots.

#### Acceptability

Most participants felt that their respective app or tag would be useful for contact tracing (25/31, 81%), though a lack of consistency between recalled interactions and Bluetooth data diminished some participants’ confidence in the technology.

The device initially failed to detect other devices, and therefore I'm worried about the efficiency of the app.

App pilot, Participant #7

I think that when it worked, it was great. There were times, such as my first day, where it didn't detect anyone even though I was well within 6 feet.

Tag pilot, Participant #15

Most participants understood how their respective device collected (27/32, 84%) and protected their data (22/32, 69%) (Table 6). With regard to social influence and study setting, there were no significant differences between pilot environments. Across both pilots, participants more frequently reported vocal support for the technology from supervisors than from peers (21/26, 81% from supervisors vs 10/27, 37% from peers; *P*=.001). The opposite was true regarding vocal concern, with participants more frequently reporting vocal concern from peers compared to supervisors (13/29, 45% from peers vs 2/25, 8% from supervisors; *P*=.003). Within the study environment, most participants felt that adequate technical assistance was available when needed (20/28, 71%), and also felt that, should the university adopt such technology, they would maintain individual agency over whether or not they used the devices (26/31, 84%).

**Table 6:** Postparticipation survey: acceptability domain.

Subdomains and questions		Total percentage agreement^a^ (N=32)^b^, % (n/N)	App percentage agreement^a^ (N=20)^b^, % (n/N)	Tag percentage agreement^a^ (N=12)^b^, % (n/N)	*P* value^c^
**Usefulness**					
	The system would be useful for contact tracing.	81 (25/31)	74 (14/19)	92 (11/12)	.22
	The app has all the functions and capabilities I expected it to have.	58 (18/31)	42 (8/19)	83 (10/12)	.02
**Coherence**					
	I understand how data collected with this system would be used for contact tracing.	84 (27/32)	80 (16/20)	92 (11/12)	.38
	I understand how this system currently protects my privacy.	69 (22/32)	65 (13/20)	75 (9/12)	.56
**Social influence**					
	Peers whose opinions I value have vocalized their support for this system.	37 (10/27)	24 (4/17)	60 (6/10)	.06
	Supervisors in my workplace have vocalized their support for this system.	81 (21/26)	83 (15/18)	75 (6/8)	.62
	Peers whose opinions I value have voiced concerns about using this system.	45 (13/29)	50 (9/18)	36 (4/11)	.47
	Supervisors in my workplace have voiced concerns about using this system.	8 (2/25)	12 (2/17)	0 (0/8)	.31
**Setting**					
	Technical assistance was available when needed.	71 (20/28)	71 (12/17)	73 (8/11)	.90
	The decision to use or not use this system will remain under my control.	84 (26/31)	79 (15/19)	92 (11/12)	.35

^a^Percentage agreement was calculated by dividing the number of Likert responses indicating agreement by the total number of Likert responses for each question.

^b^Some questions were not answered by all participants; exact counts of agreement and total responses are shown in parentheses for each question.

^c^*P* values obtained using tests of proportions to evaluate differences between pilots.

#### Adherence

There was no difference between pilots in overall adherence rates based on self-reported percentages of shifts during which the study device was carried (mean 87%) (Table 7), although participants in the tag pilot more commonly reported that their study device was convenient to carry than did participants from the app pilot (tag pilot: 11/12, 92% vs app pilot: 11/20, 55%; *P*=.03). While some participants from the app pilot reported leaving the device at home (2/13, 15%), participants from both pilots reported that the most common reason for not carrying the devices was forgetting it at a workstation (17/23, 74%). App pilot participants also reported inabilities to carry the study device into certain lab environments (app pilot: 5/13, 38% vs tag pilot: 0/10, 0%; *P*=.03), while tag pilot participants reported that charging the device interfered with adherence (tag pilot: 3/10, 30% vs app pilot: 0/13, 0%; *P*=.03).

Many participants from the app pilot used the free-text response to note the inconvenience of carrying an additional phone and suggested that a smaller device be used. A minority suggested that they be allowed to download the tracing app directly on their personal phones. Gender-specific difficulties in carrying the app pilot study phone were also noted by 1 participant, while a separate participant from the tag pilot noted the relative ease of carrying the tag.

The only problem I found with this [study phone] is that it is big. For women it just does not fit in the front pocket of the jeans and in the summer, you are not wearing a jacket under your lab coat. So, the only place left is the pocket of the jeans in the back. And that is a bit uncomfortable when you sit down, or you are scared it might fall out. I also do not feel good putting it in the pockets of my lab coat because I consider them “dirty” and I do not want to have lab dirt in my home, or touch it without gloves. So, it would be much more convenient if it would be a bracelet or a watch or something around those lines.

App pilot, Participant #12

The shape of [the tag] is pretty clunky to carry around, but as long as you wear pants with pockets it's easy enough to just wear in your back pocket.

Tag pilot, Participant #16

The vast majority of participants from the app pilot reported that they would be more likely to carry a Bluetooth device if it were smaller than a phone (19/20, 95%), while no participants from the tag pilot (0/12, 0%) agreed that increasing the size of the tag would increase adherence (*P*<.001), indicating an overall preference for smaller devices.

**Table 7:** Postparticipation survey: adherence domain.

Questions			Total percentage agreement^a,b^ (N=32)^c^, % or % (n/N)		App percentage agreement^a,b^ (N=20)^c^, % or % (n/N)		Tag percentage agreement^a,b^ (N=12)^c^, % or % (n/N)		*P* value^d^
Over the course of the 2-week study period, for what proportion of your total work shifts did you have the device either on you or within arms’ reach?			87^e^		91^e^		81^e^		.06
The device was convenient to carry with me throughout my work shifts.			69 (22/32)		55 (11/20)		92 (11/12)		.03
**How did you carry the device with you throughout your workday? (tag only)**									
	Pocket	N/A^f^		N/A		92 (11/12)		N/A	
	Bag	N/A		N/A		0 (0/12)		N/A	
	Belt/lanyard	N/A		N/A		8 (1/12)		N/A	
	Left at workspace	N/A		N/A		8 (1/12)		N/A	
**What were the most common reasons why you would not carry the device with you during a work shift?**									
	Forgot at home	9 (2/23)		15 (2/13)		0 (0/10)		.19	
	Intentionally left at home	0 (0/23)		0 (0/13)		0 (0/10)		N/A	
	Forgot at desk/workstation	74 (17/23)		69 (9/13)		80 (8/10)		.56	
	Intentionally left at desk/workstation	9 (2/23)		15 (2/13)		0 (0/10)		.19	
	Unable to carry it into certain lab environments	22 (5/23)		38 (5/13)		0 (0/10)		.03	
	Left it to charge	13 (3/23)		0 (0/13)		30 (3/10)		.03	
I would be more likely to carry the device with me if it were smaller (for instance, the size of a thumb drive that could be attached to a lanyard). (app only)			N/A		95 (19/20)		N/A		<.001
I would be more likely to carry the tag with me if it were larger (for instance, the size of a phone). (tag only)			N/A		N/A		0 (0/12)		<.001

^a^Unless otherwise specified.

^b^Percentage agreement was calculated by dividing the number of Likert or binary responses indicating agreement by the total number of responses for each question.

^c^Some questions were not answered by all participants; exact counts of agreement and total responses are shown in parentheses for each question.

^d^*P* values obtained by tests of proportions for differences in percentage agreement and by unpaired *t* tests for differences in means.

^e^Mean response.

^f^N/A: not applicable.

## Discussion

### Principal Findings and Implications

Incomplete vaccine uptake [32,33] and potential for breakthrough transmission due to new variants [34] suggest that contact tracing will remain an important tool in the ongoing response to COVID-19. However, its use thus far in the pandemic has revealed many challenges to scaling-up traditional contact tracing [40-43] and identified a need to improve upon existing methods. Digital contact tracing tools offer many opportunities to improve the impact of contact tracing [44], and increasing our understanding of how different technologies may be applied for this purpose is critical. In our dual-pilot evaluation of 2 novel contact tracing technologies, we found that Bluetooth contact tracing was perceived as appropriate to the majority of study participants, adherence to device carrying was high, and participants were largely satisfied with their experiences. However, most participants still reported concerns about privacy, and both technologies encountered occasional technical glitches. Importantly, we also found that the tag-based device was easier to carry and had superior sensitivity and specificity. These increased performance metrics may have been due to differences between the Bluetooth signal strength settings of the technologies or in how participants carried the different study devices, as reflected in the postparticipation survey.

Our findings are similar to a recent study [45] that compared a Bluetooth mobile app to a wearable, radio frequency-based, real-time locator device within a health care setting. The researchers found the wearable device to be superior to Singapore’s “TraceTogether” app with regard to sensitivity and specificity, and also found that the app’s performance was worse on iPhones compared to Android devices. In a similar study, a wearable device was compared to electronic medical record-assisted tracing and was again found to be superior [46]. Our study builds upon these findings by evaluating similar app-based technology in a new university setting, while also comparing it directly to a novel Bluetooth tag device, rather than a radio frequency-based device.

Although most proximity-based contact tracing technologies offer similar benefits, such as the ability to identify unknown contacts or customize detection thresholds based on evolving knowledge of transmission dynamics [47], different approaches (eg, app vs carriable device) offer certain additional benefits and drawbacks. Below, we discuss key differences while paying heed to the importance of context. While traditional contact tracing focuses on community and population transmission, COVID-19 has led many closed-door environments, such as workplaces, schools, universities, and hospitals, to conduct contact tracing independently from, or in partnership with, local public health systems [48,49]. The differences between community tracing and closed-door tracing are important when comparing app-based and tag-based systems, as different contexts are often coupled with different funding capacities, thresholds for acceptable uptake of tracing technology, and user privacy concerns.

Deploying Bluetooth tracing technology to communities or populations at large is likely only feasible using an app-based system. App-based tracing technologies, such as those developed by Apple and Google, have already been deployed throughout the globe [16], including in many US states [26], with relatively little cost to distribution beyond social marketing. Meanwhile, it would not be logistically or financially feasible to deploy a similar number of tag devices throughout the population, as each tag costs approximately US $10. Furthermore, while updating apps is relatively seamless, updating hardware poses a greater challenge, as we encountered in this study when we discovered a defect in our tag cases. Despite these potential drawbacks, tags and similar approaches may be more feasible in closed-door environments that have available funds to spend on the protection of a much smaller population.

Acceptable thresholds for uptake may also differ between environments, making the logistical concerns noted above more or less important across different settings. Public health officials in many countries are often hesitant or unable to mandate participation in health interventions, as demonstrated with mask policies in response to COVID-19 [50]. Public health programs also frequently lack funding to properly incentivize participation. As a result, population-wide uptake of app-based technology for tracing will likely always be limited. Closed-door environments, on the other hand, may face greater pressure to standardize and ensure the safety of all staff, students, or workers, and therefore may prioritize, or mandate, comprehensive uptake, as demonstrated by many universities requiring vaccination for all students [51]. However, reaching such a high uptake of digital contact tracing without diminishing individual agency or ignoring privacy concerns poses a challenge.

Privacy concerns are often related to the types of information collected as well as the organization or government collecting the data [23,24], and may be heightened in the context of a pandemic [52]. Notably, our study participants felt that using Bluetooth data for tracing was more appropriate than GPS or Wi-Fi data. While technologies, such as blockchain, may increase the security of app-based approaches [53] and further reduce the risks of data leakage, effectively communicating such methods and establishing trust with potential users may remain difficult as long as data collection relies on personal devices, as reflected by our participants’ preferences against using apps on their phones. This provides several arguments for shifting data collection away from personal devices and onto organization-owned tracing tags when possible. First, the tag-based system offers users in closed-door environments the opportunity to participate in contact tracing without requiring data collection on their phones. While our study still relied on an app to sync the tag’s data, the provision of “syncing stations” throughout closed-door environments could eliminate the need for an app entirely and further reduce concerns about leakage of personal phone data. Second, the use of organization-owned tags addresses concerns about governments or third-party companies accessing personal data [23,52], which was reflected in our participants’ preferences against third party apps. Ultimately, these features offer the potential to reduce privacy concerns and increase uptake within closed-door environments.

There are several key strengths to this study, including the use and evaluation of novel technologies developed directly in response to the COVID-19 pandemic. Second, the setting in which the study was conducted is typical of some other environments, in particular schools and universities, that have struggled to perform contact tracing throughout the pandemic, making this study increasingly relevant to public health practitioners or researchers operating in similar environments. Lastly, the use of mixed methods, including sensitivity and specificity estimations, survey analyses, and qualitative analysis, allowed us to triangulate our findings and present a layered evaluation of the technologies’ performance metrics as well as the users’ experiences.

There are also several important limitations in this study. First, the sample size was relatively small, increasing the risk of type II errors. Second, the recruitment of different labs and participants for each pilot created some uncertainty about the mechanisms driving the observed differences in Bluetooth performance metrics and user experiences or perceptions. However, the lack of significant differences in survey responses regarding setting and social influences, and the baseline similarities in lab environments selected for the study minimize this risk. Third, the lack of a true “gold standard” measurement for close contact interactions introduces the potential for bias in the estimations of sensitivity and specificity. In particular, recall bias may have led to misreporting of self-report contacts, and the lack of precise measurements for the length of self-report interactions between participants may have introduced additional uncertainties. However, participants’ daily review and validation of contact interactions likely minimized the potential for recall bias, which would have been more severe if the data were collected less frequently. Furthermore, these potential biases likely affected each pilot similarly, which lessens the degree to which such biases may have affected the comparisons between pilots. Fourth, based upon the participant-initiated method of qualitative data collection (optional free-text box vs traditional interview queries), it is doubtful that meaning saturation [54] was achieved and likely that themes would have been better explicated and perhaps more abundant if a traditional approach to qualitative interviewing had been used. Nonetheless, the study provides preliminary evidence about the relative merits of the 2 technologies that can inform larger studies in the future. Fifth, demographic data were not collected from participants at the time of recruitment, limiting our ability to evaluate differences across participant characteristics. Considering the small sample size and short timeframe of the pilots, we lacked statistical power to evaluate differences across participant characteristics and therefore did not include this as a study goal. Last, the relative homogeneity of the study sample may limit the generalizability of our findings to other nonuniversity contexts, which may feature differences in behavior, familiarity with technology, and/or attitudes [55].

### Conclusion

As vaccine uptake remains noncomprehensive and new variants appear, contact tracing will remain a pillar of the public health response to COVID-19. Increasing the efficiency of contact tracing through adoption of technologies, such as those evaluated here, may improve its impact and ability to prevent or control outbreaks. This is among the first studies to directly evaluate the performance metrics of novel Bluetooth technologies when used for COVID-19 contact tracing in conjunction with evaluations of user experiences. Our participants found Bluetooth-assisted tracing to be appropriate, and we noted several key differences between app-based and tag-based approaches. The benefits of the app-based system include its low cost and theoretical ease of mass distribution, and the drawbacks include increased privacy concerns of users. The benefits of the tag system include its superior sensitivity and specificity, the ease of carrying the tag, and the potential to alleviate user privacy concerns, and the drawbacks include its reliance on hardware that may be less feasible to deploy in certain settings.

## Appendix

Multimedia Appendix 1 Screenshot of the app pilot mobile app.

Multimedia Appendix 2 Bluetooth device (“tag”) used in the tag pilot.

Multimedia Appendix 3 Screenshot of the tag pilot mobile syncing app.
