# Supplementary material for: Pilot Evaluations of Two Bluetooth Contact Tracing Approaches on a University Campus: Mixed Methods Study
Source: JMIR Form Res. 2021 Oct 28;5(10):e31086. doi: 10.2196/31086 (PMC8555945; doi:10.2196/31086)
Supplement: Multimedia Appendix 1 [file formative_v5i10e31086_app1.docx]

**Multimedia Appendix 1.** Screenshot of the app pilot mobile app.


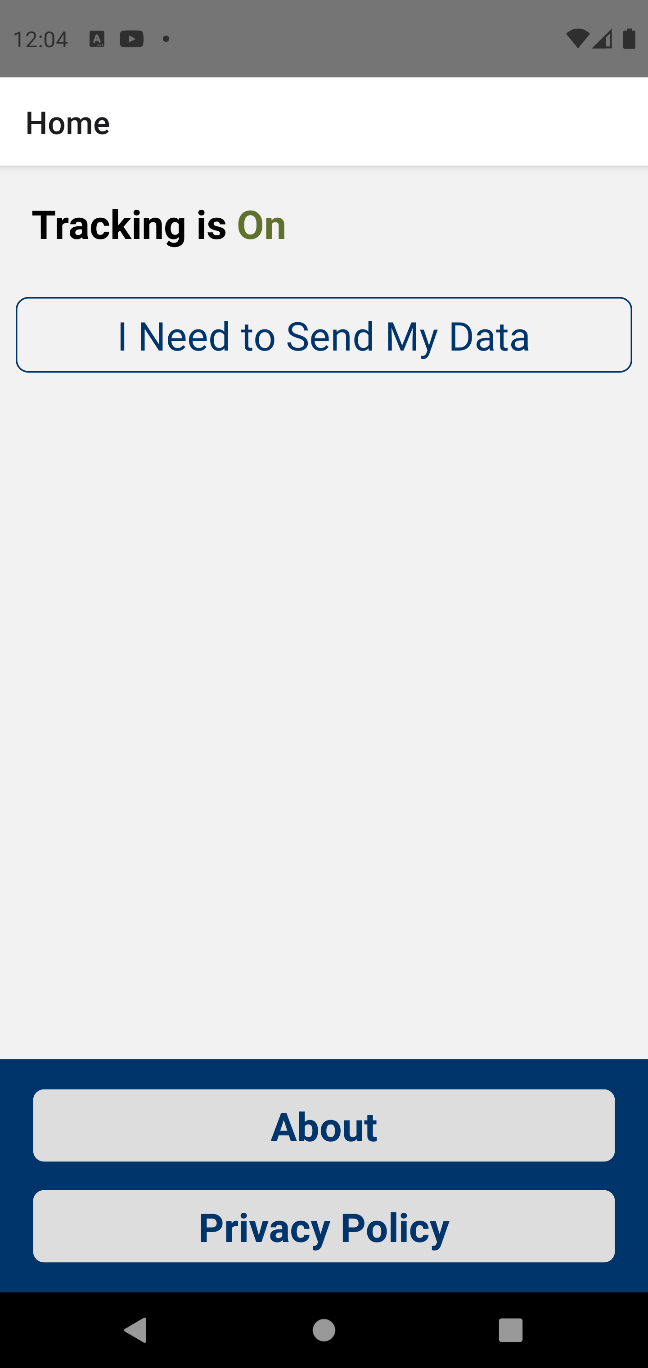


Legend: This image contains a screenshot of the contact tracing mobile app used in the app pilot.
