# Supplementary material for: Pilot Evaluations of Two Bluetooth Contact Tracing Approaches on a University Campus: Mixed Methods Study
Source: JMIR Form Res. 2021 Oct 28;5(10):e31086. doi: 10.2196/31086 (PMC8555945; doi:10.2196/31086)
Supplement: Multimedia Appendix 2 [file formative_v5i10e31086_app2.docx]

**Multimedia Appendix 2.** Bluetooth device (“tag”) used in the tag pilot.


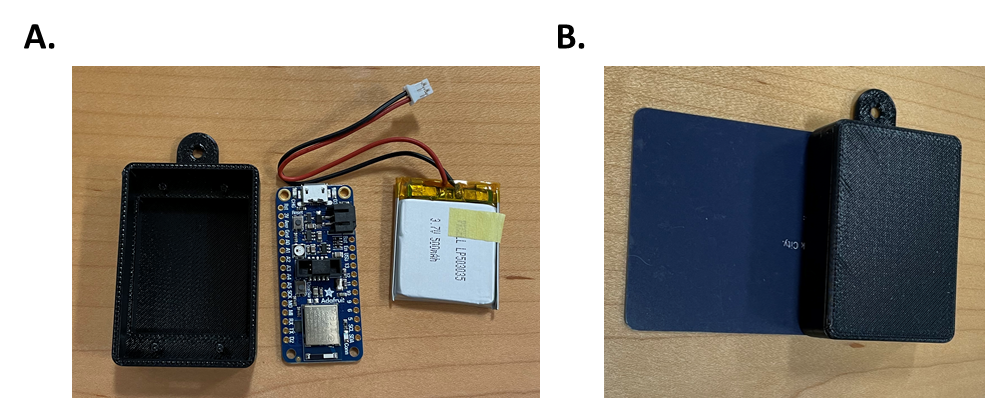


Legend: These images display the tag components (A) and the assembled tag alongside a typical gift card for size comparison (B). Tag dimensions were roughly 6 cm x 3.8 cm x 1.6 cm.
