# Supplementary material for: Pilot Evaluations of Two Bluetooth Contact Tracing Approaches on a University Campus: Mixed Methods Study
Source: JMIR Form Res. 2021 Oct 28;5(10):e31086. doi: 10.2196/31086 (PMC8555945; doi:10.2196/31086)
Supplement: Multimedia Appendix 3 [file formative_v5i10e31086_app3.docx]

**Multimedia Appendix 3.** Screenshot of the tag pilot mobile syncing app.


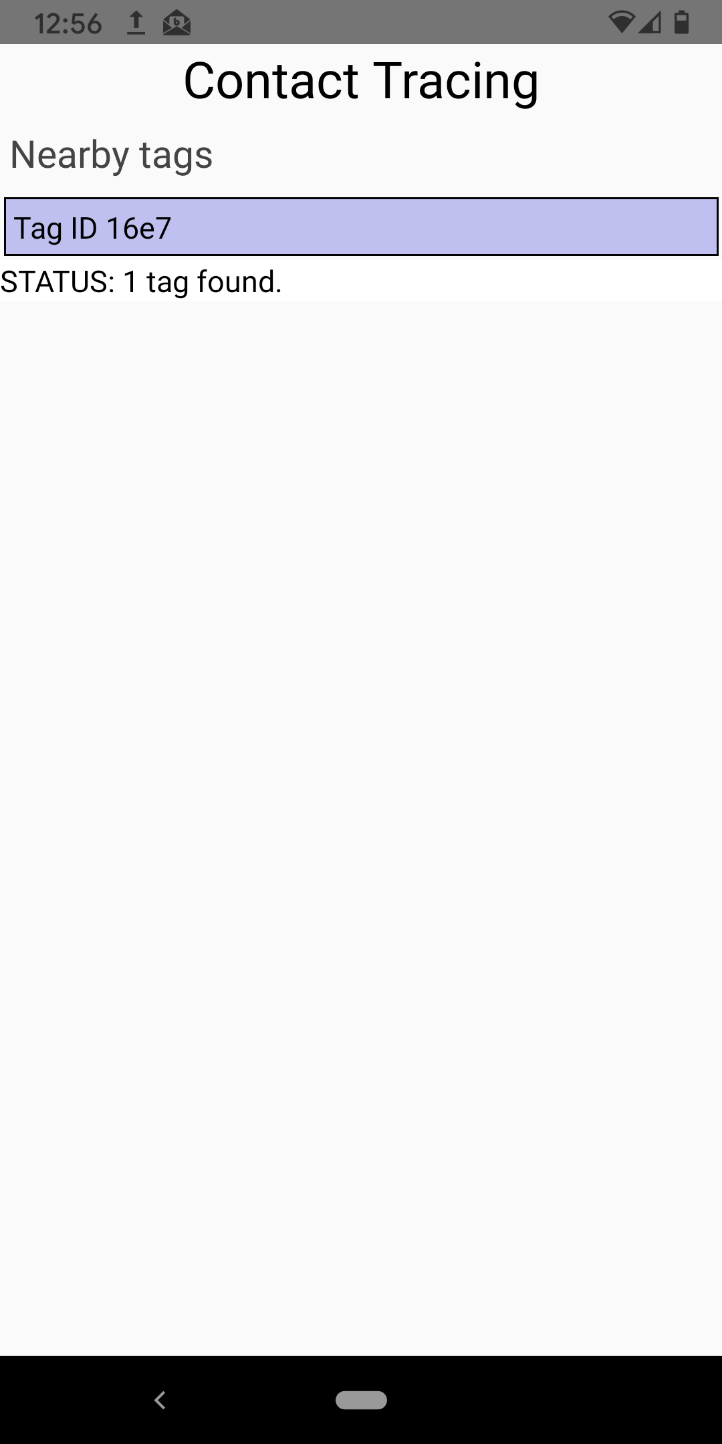


Legend: This image displays a screenshot of the syncing app used by participants in the tag pilot. To sync the data from their tag to the central servers, participants held the tag near the phone until it was detected by the mobile app (as shown in the blue stripe above), and then “pressed” on the Tag ID shown.
